# TUmor-volume to breast-volume RAtio for improving COSmetic results in breast cancer patients (TURACOS); a randomized controlled trial

**DOI:** 10.1186/s12885-017-3280-y

**Published:** 2017-05-17

**Authors:** M. Lagendijk, E. L. Vos, A. H. J. Koning, M. G. M. Hunink, J. P. Pignol, E. M. L. Corten, C. de Monye, C. H. M. van Deurzen, J. H. van Dam, W. W. Vrijland, C. M. E. Contant, C. Verhoef, W. van Lankeren, L. B. Koppert

**Affiliations:** 1 Department of Oncological Surgery, Erasmus MC Cancer Institute, DHA-102, PO Box 5201, 3008 AE Rotterdam, The Netherlands; 2 Department of Bioinformatics, Erasmus MC, Rotterdam, the Netherlands; 3 Department of Epidemiology and Department of Radiology, Erasmus MC, Rotterdam, the Netherlands; 4 Center for Health Decision Science, Harvard T.H. Chan School of Public Health, Boston, MA USA; 5 Department of Radiotherapy, Erasmus MC, Rotterdam, the Netherlands; 6 Department of plastic and reconstructive surgery, Erasmus MC, Rotterdam, the Netherlands; 7 Department of Radiology, Erasmus MC, Rotterdam, the Netherlands; 8 Department of pathology, Erasmus MC, Rotterdam, the Netherlands; 9 Department of surgery, Havenziekenhuis, Rotterdam, the Netherlands; 10 Department of surgery, Franciscus Gasthuis, Rotterdam, the Netherlands; 11 Department of surgery, Maasstad ziekenhuis, Rotterdam, the Netherlands

## Abstract

**Background:**

Cosmetic result following breast conserving surgery (BCS) for cancer influences quality of life and psychosocial functioning in breast cancer patients. A preoperative prediction of expected cosmetic result following BCS is not (yet) standard clinical practice and therefore the choice for either mastectomy or BCS is still subjective. Recently, we showed that tumour volume to breast volume ratio as well as tumour location in the breast are independent predictors of superior cosmetic result following BCS. Implementation of a prediction model including both factors, has not been studied in a prospective manner. This study aims to improve cosmetic outcome by implementation of a prediction model in the treatment decision making for breast cancer patients opting for BCS.

**Methods/design:**

Multicentre, single-blinded, randomized controlled trial comparing standard preoperative work-up to a preoperative work-up with addition of the prediction model. Tumour volume to breast volume ratio and tumour location in the breast will be used to predict cosmetic outcome in invasive breast cancer patients opting for BCS. Three dimensional (3D)-ultrasonography will be used to measure the tumour volume to breast volume ratio needed for the prediction model. Sample size was estimated based on a 14% improvement in incidence of superior cosmetic result one year after BCS (71% in the control group versus 85% in the intervention group). Primarily cosmetic outcome will be evaluated by a 6-member independent panel. Secondary endpoints include; (1) patient reported outcome measured by BREAST-Q, EORTC-QLQ-C30/BR23 and EQ-5D-5 L (2) cosmetic outcome as assessed through the BCCT.core software, (3) radiation-induced reaction (4) surgical treatment performed, (5) pathological result and (6) cost-effectiveness. Follow-up data will be collected for 3 years after surgery or finishing radiotherapy.

**Discussion:**

This randomized controlled trial examines the value of a preoperative prediction model for the treatment-decision making. It aims for a superior cosmetic result in breast cancer patients opting for BCS. We expect improvement of patients’ quality of life and psychosocial functioning in a cost-effective way.

**Trial registration:**

Prospectively registered, February 17th 2015, at ‘Nederlands Trialregister - NTR4997’.

**Electronic supplementary material:**

The online version of this article (doi:10.1186/s12885-017-3280-y) contains supplementary material, which is available to authorized users.

## Background

For early stage breast cancer patients the goal of therapy is to ensure both local control and breast preservation with an optimal cosmetic outcome. The current standard of care is breast-conserving surgery (BCS) followed by adjuvant whole breast irradiation [1–3]. Large randomised clinical trials in the 80’s have shown that this treatment is equivalent, in terms of overall and breast-cancer specific survival, to a mastectomy [4]. The frequency of BCS performed is estimated at an annual rate of 60% in the Netherlands [5]. Oncoplastic surgery, an operation performed jointly by a plastic surgeon and a breast-cancer surgeon specialist, is currently more frequently performed with the goal of obtaining the best cosmetic outcome possible. At time of diagnosis, however, the treatment decision whether to perform a mastectomy (with or without a breast reconstruction) or BCS is often subjective. Unfavourable cosmetic outcome following BCS is significantly associated with decreased quality of life and psychosocial functioning [6, 7]. Poor cosmetic outcome following BCS is reported in up to 30% of breast cancer patients [8–11]. Pre-operative knowledge of the expected cosmetic outcome would thus be a welcome treatment decision aid.

As previously studied by our group, independent factors for the prediction of cosmetic outcome in BCS are tumour location and tumour volume to breast volume ratio. This volume ratio was obtained through 3-D visualisation of breast MRI images (10). Based on these factors a prediction model is made predicting the expected cosmetic outcome for BCS. The prediction model could aid in the treatment decision by differentiation of patients with an expected favourable cosmetic outcome and thereby improve cosmetic outcome and patients’ quality of life.

Within this randomised trial participant will undergo an additional 3-D ultrasonography of the affected breast. Ultrasonography has a broad clinical applicability in breast cancer patients and is not dependent on the use of ionizing radiation. Measurements, obtained by the Automated Breast Volume Scanner (ABVS) and 3-D ultrasonography (3-D US) have previously been compared to MRI and histopathological tumour size with good agreement [12, 13]. An additional validation compared volume measurements of the3-D US to those measured by histopathological and 3-D MRI. Both breast and tumour volume showed high agreement (unpublished data).

This study aims to improve cosmetic outcome following BCS by using a preoperative prediction model based on 3-D ultrasonography.

### Objective

The objective of this randomized controlled trial is to compare cosmetic outcome following a standard preoperative work-up by that of the preoperative prediction model. The hypothesis is that the addition of the preoperative prediction model aids in the treatment-decision making and therefore improves cosmetic outcome and quality of life in patients opting for BCS.

## Methods/design

### Trial design

**Fig. 1:**
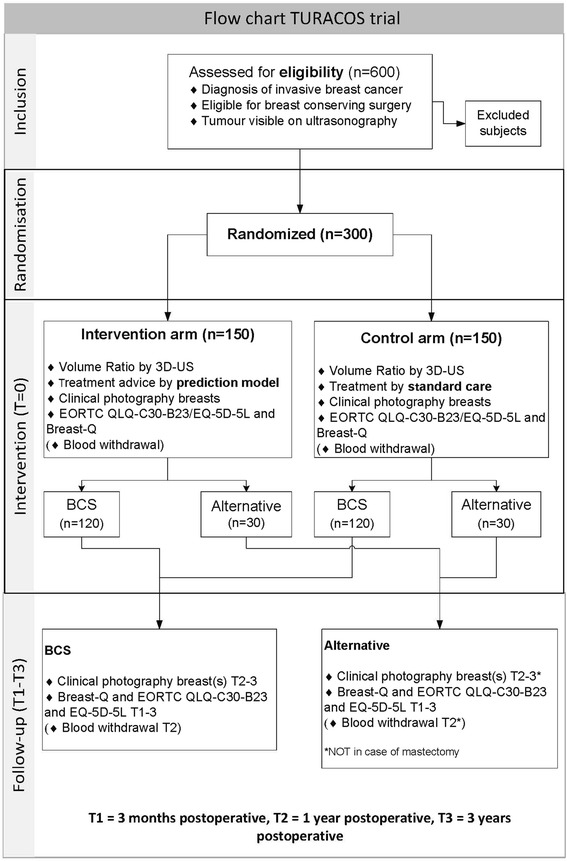
Flow chart TURACOS trial

This single-blinded, multicentre, randomised controlled trial targets women with the diagnosis of primary breast cancer. Patients will randomly be assigned (1:1) to either the intervention or control group after written informed consent is given. The study is in compliance with the Helsinki declaration. Ethical approval has been granted by the Institutional Review Board of the Erasmus University Medical Centre, Rotterdam, the Netherlands (reference-number: MEC-2013-360). This trial is registered before the start of the inclusion on February 17th, 2015 (NTR 4997). Figure 1 present a flow-diagram of the trial design.

### Participants

Women with pathologically confirmed primary invasive breast cancer (cT1–3) that are eligible and opt for BCS will be included. Additional inclusion and exclusion criteria are presented in Table 1.

**Table 1:** Inclusion and exclusion criteria

Inclusion criteria	Exclusion criteria
Female	Recurrent breast cancer ipsilateral
Aged >18 years	Previous radiation therapy ipsilateral breast
Primary breast cancer	
Pathologically proven breast cancer TI-III	
Eligible for breast conserving surgery (BCS)	

### Interventions

#### Volumetric measurements

All patients will receive an additional ultrasound performed by the Automated Breast Volume Scanner (ABVS– ACUSON S2000™ ABVS, Siemens Medical Solutions, Inc., Mountain View, CA) available at the department of Radiology, Erasmus MC Cancer Institute [14]. A standardized scanning technique is used where 3 or 5 scans are performed (i.e. anterior-posterior, lateral and medial view or anterior-posterior, upper lateral-, lower lateral-, upper medial- and lower medial-quadrant). Data from the ABVS is visualised by the ‘V-scope’ software (department of Bioinformatics, Erasmus MC) through an interactive 3-D image on the desktop system. Volume measurements are performed using a tracking system and a wireless joystick [15].

#### Prediction model

The tumour to breast volume ratio and the location of the tumour in the breast are used in the prediction model to calculate the chance of superior cosmesis in case of BCS. If the probability exceeds the predefined threshold primary BCS is advised. If the probability is below the predefined threshold an alternative treatment will be advised (i.e. oncoplastic BCS, mastectomy with or without a breast reconstruction or neo-adjuvant chemotherapy). The threshold is defined by modelling the differences in quality of life utilities between the different surgical strategies and the associated cosmetic results.

#### Pre-operative work-up

For all randomized patients the following characteristics will be collected (if applicable): age, body mass index (BMI), comorbidity, previous operations, smoking status, hormonal status, tumour morphology, TNM stadium, post-operative complications, radiotherapy details, chemotherapy details and hormonal therapy details.

Preoperatively all patients will be asked to complete three questionnaires. This includes the ‘Breast Q – preoperative modules’ [16], the ‘European Organisation of research and Treatment of Cancer quality of life questionnaire (EORTC-QLQ-C30 version 3 and EORTC-QLQ-B23 version 1)’ and ‘Euro-QoL 5D-5 L questionnaire’ (EQ-5D-5 L-version 2). All patients will preoperatively be discussed in multidisciplinary consultation. For the intervention group, the optimal treatment based on the results of the prediction model will be taken into consideration. All patients will be blinded for the result of the prediction model. Preoperative photographs will be taken by a professional medical photographer. These photographs include the 1) face-view position with the arms down, 2) face-view with arms in the side and 3) lateral view (90°) off the affected breast. The face view photographs will cover the area of chin to the umbilicus protecting the subject’s privacy and anonymity.

#### Follow-up

Three questionnaires will be obtained at all follow-up visits (i.e. ‘BREAST-Q postoperative modules’, the EORTC-QLQ-C30/B23 and the EQ-5D-5 L. Follow-up visits will be scheduled at 3 months (T1), 1 year (T2), and 3 year (T3) postoperatively. Photographs will be obtained both 1 and 3 year following BCS.

### Outcomes

The outcome measures are summarized in Table 2.

**Table 2:** Assessments and used instruments with timeframe indication

Outcome(s)	Instrument(s)	T0	T1	T2	T3
Primary endpoint					
Cosmetic result	Evaluation clinical photos by panel - ‘Erasmus MC panels’ questionnaire’	x		x	x
Secondary outcome					
Cosmetic result/satisfaction with breast assessed by patient	Breast Q - preoperative module	x			
	Breast Q - postoperative module		x	x	x
Patients’ Quality of life	EORTC QLQ-C30/B23 questionnaire	x	x	x	x
	EQ-5D-5 L questionnaire	x	x	x	x
Cosmetic result BCCT.core	BCCT.core software (INESC Porto Breast Research Group)	x		x	x
Radiation-induced reaction	RTOG/EORTC			x	x
Surgical strategy performed	Performed surgery/surgeries				x
Pathological result	Data of pathology reports				x
Cost-effectiveness	Quality-Adjusted Life Year (QALY) for direct and indirect costs				x

T0 = first visit outpatient clinic, T1 = 3 months postoperative, T2 = 1 year postoperative, T3 = 3 years postoperative

#### Primary outcome

The cosmetic outcome evaluation will be performed by a 6-member, independent panel using the photographs. Each panel consist of 1) a plastic surgeon/breast cancer surgeon, 2) a general practitioner, a medical doctor, 3) a radiation oncologist, 4) a layperson and 5) a breast cancer survivor. Cosmetic outcome will be evaluated using a previously reported in-house developed questionnaire containing 11 aggregating previous recommendations [17] (see Additional file 1 A ‘Erasmus MC panels’ questionnaire’). Answers are scored on a four point Harvard cosmetic scale: 0 = ‘excellent’, 1 = ‘good’, 2 = ‘moderate’, and 3 = ‘bad’ [18].

*Secondary outcome(s).*

1) Patient reported outcome is measured by the ‘BREAST-Q’, ‘EORTC-QLQ-C30/BR23’ and ‘EQ-5D-5 L’ questionnaires (T0–3).

2) Cosmetic outcome assessed by the ‘BCCT.core software’ - INESC Porto Breast Research group [19] based on medical photographs (T0–2-3).

3) Radiation reaction scored by the ‘Radiation Therapy Oncology Group/European Organization for Research and Treatment of Cancer’ – ‘RTOG/EORTC’ (T2–3) [20].

4) Surgeries performed (T3).

5) Pathological results (T3).

6) Cost-effectiveness*;* direct costs will include the pre-operative care together with the costs for (surgical) treatment. Indirect costs will generally include adjuvant operation(s) if applicable and costs of outpatient clinic visits or hospitalisation (T4).

### Data collection and statistical analysis

#### Primary study parameter

Cosmetic outcome is calculated by obtaining the mean score for all panel members. The score per panel member is based on the mean score off the 11 scored items combined. Subsequently, the mean panel evaluation will be dichotomized by defining a mean of <1.5 as superior and a mean of >1.5 as inferior. The primary outcome is the incidence of a superior cosmetic outcome in both arms. Difference will be evaluated by using the Chi-square analysis (if normally distributed) or the non-parametric Kruskal-Wallis test (if not normally distributed).

#### Secondary study parameters

Cosmetic result assessed by the patient (BREAST-Q), cosmetic evaluation through BCCT.core and radiation-induced reaction (RTOG/EORTC) will be analysed by comparing the intervention and control group. For normally distributed categorical data, the Chi-Square test will be used and the Kruskal Wallis test if not normally distributed. Continues variables as the Q-score will be analysed making use of the Student T-test if normally distributed or Mann-Whitney U test if not normally distributed. Pathological result will be analysed by comparing the percentages of incomplete tumour excisions and mean lumpectomy specimen size between the two study arms. The secondary study parameters will be analysed based on the intention-to-treat principle (i.e. including the patients treated with mastectomy). Surgical strategies performed will be analysed by comparing the percentages of the different types of surgery performed by using a Chi-square analysis if normally distributed or Kruskal-Wallis test if not normally distributed. Patients’ quality of life, conducted through the EORTC questionnaire/Euro-QoL and BREAST-Q questionnaires will be presented in a quantitative manner. Cost-effectiveness will be calculated by Markov modelling using ‘Quality-Adjusted Life Years’ (QALY’s).

### Sample size

In our previously performed retrospective study the incidence of a superior cosmetic result, without intervention, was 71%. An improvement of superior cosmetic outcome to 85% was considered clinically relevant. Expected clinical relevant difference is a 14% improvement in the incidence of a superior cosmetic result as evaluated by an independent panel. The sample size calculation is based on comparing two proportions in independent groups by the Chi-Square test. With 80% power and 5% significance level we need a study population of 240 patients (120 in each arm). There is a possibility that even after randomization the patient can still undergo mastectomy instead of BCS. We expect this for less than 25% patients in each arm, therefore we aim to include 300 patients in total (150 in each arm).

## Discussion

The literature provides limited predictive factors for an expected favourable cosmetic outcome in breast cancer patients opting for BCS. To objectively device a tailor-made treatment plan this study makes use of a prediction model [10]. This study aims to provide level 1B evidence for the use of a preoperative prediction model for clinical decision making to improve cosmetic results in patients opting for BCS.

Following the inclusion of the first 30 patients (10%) the patients’ experiences tell us that the study is well accepted and appreciated. It is however of great importance that the study is discussed by the treating surgeon at the first or second consultation at the outpatient clinic. By the start of inclusion up to 40% of the approached patients declined participation. This was mainly based on too much burdening in the preoperative phase and an inadequate introduction of the study. If the treating surgeon introduces the study and explains the importance of the study in the preoperative phase the acceptance for participation is higher. With the allocation of multiple including centre’s in the region of Rotterdam the inclusion rate has adequately improved with an ongoing high acceptance of participating patients.

Preoperative assessment of patients’ quality of life and satisfaction with their breast is currently lacking in the literature available. Only few trials have combined postoperative cosmetic outcome measurements by panel or software with patient reported outcome measures (PROMs) [21–23]. Especially an evaluation of cosmetic outcome through time following breast surgery is scarce [21]. By preoperatively collecting aesthetics and PROMs a reliable understanding of the relationship between cosmetic results and self-image or quality of life is gained. Comparing overall health-related quality of life (EORTC-QLQ-C30/B23 and EQ-5D-5 L) and treatment- or surgery specific outcomes (BREAST-Q) gives a better understanding between overall and disease-specific quality of life [16, 24, 25]. With this knowledge, future treatment decision making and cosmetic outcome evaluation can possibly be based on PROMs. To adequately study cosmetic outcome and their relationship to PROM’s, standardized, reproducible and easily available tools are needed [18, 26]. Comparing two different panel evaluations within 68 patients following BCS our group found almost perfect inter- and intra-observer agreement. Interclass correlation coefficient showed *R* = 0.93, *R* = 0.9 respectively for the inter- and intra-observer agreement [unpublished data]. When comparing trials differences in panel evaluations found are based on panel size and the use of layperson versus experts [23, 27, 28]. Moreover multiple and unstandardized questionnaires are used to obtain cosmetic outcome; making a comparison between different trials difficult. Based on previous recommendations of Cardoso et al. our current study uses a questionnaire concerning the different aspect of cosmetic outcome when evaluating the breast by panel members [17]. The BCCT.core software is known to evaluate asymmetry, skin colour difference(s) and scar features based on the situation of two identical breasts [29–31]. In line with previous literature our group found a moderate agreement (unpublished data) between panel and BCCT core evaluation [22, 23]. An independent 6-member panel will therefore assess cosmetic outcome as our primary outcome. In summary this study aims to improve cosmetic outcome and quality of life through the implementation of a preoperative prediction model for breast cancer patients opting for BCS.

## Additional file

Additional file 1: Scoring list cosmetic outcome (panel evaluation). (JPEG 218 kb)

## Abbreviations

3-D: Three-dimensional
ABVS: Automated Breast Volume Scanner
BCS: Breast Conserving Surgery
BCCT.core: Breast Cancer Conservative Treatment. cosmetic result
BRA: Breast Retraction Assessment
DSMB: Data Safety Monitoring Board
EORTC-QLQ: European Organisation of Research and Treatment of Cancer quality of life questionnaire
EQ-5D-5 L: Euro-QoL-5D-5 L
QALY’s: Quality-Adjusted Life Years
RTOG/EORTC: Radiation Therapy Oncology Group/European Organization for Research and Treatment of Cancer
US: Ultrasonography
