# Supplementary figures and images for: TUmor-volume to breast-volume RAtio for improving COSmetic results in breast cancer patients (TURACOS); a randomized controlled trial
Source: BMC Cancer. 2017 May 17;17:336. doi: 10.1186/s12885-017-3280-y (PMC5436425; doi:10.1186/s12885-017-3280-y)

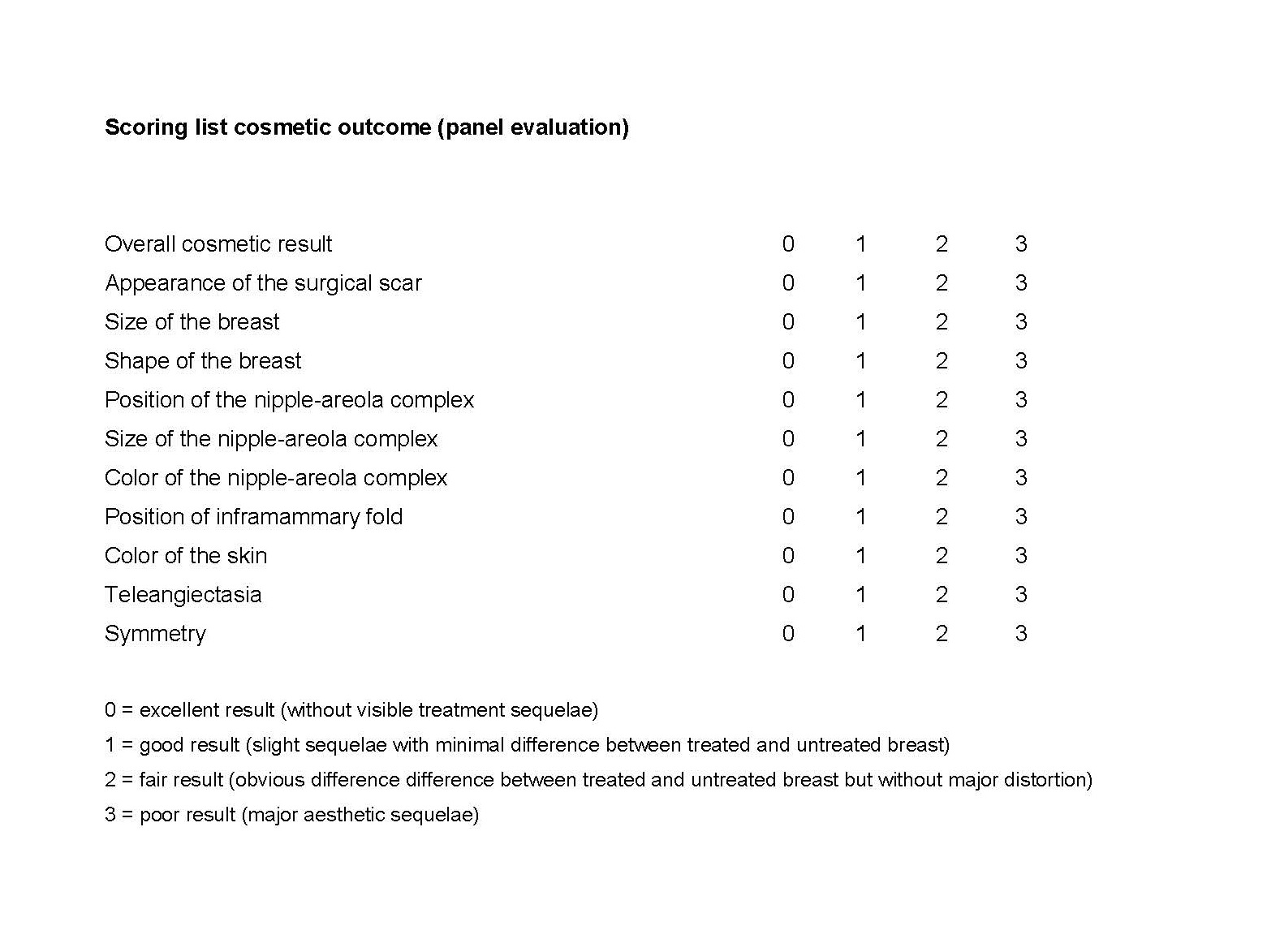

Supplement: Additional file 1: — Scoring list cosmetic outcome (panel evaluation). (JPEG 218 kb) [file 12885_2017_3280_MOESM1_ESM.jpg]
